# Supplementary material for: Causal Relationship between Aging and Anorexia Nervosa: A White-Matter-Microstructure-Mediated Mendelian Randomization Analysis
Source: Biomedicines. 2024 Aug 16;12(8):1874. doi: 10.3390/biomedicines12081874 (PMC11351342; doi:10.3390/biomedicines12081874)
Supplement: Supplementary file 1 [file biomedicines-12-01874-s001.zip › Supplementary Figure/Figure S4/Singe SNP plot of MR analysis for TL to ubm-b-1655.pdf]

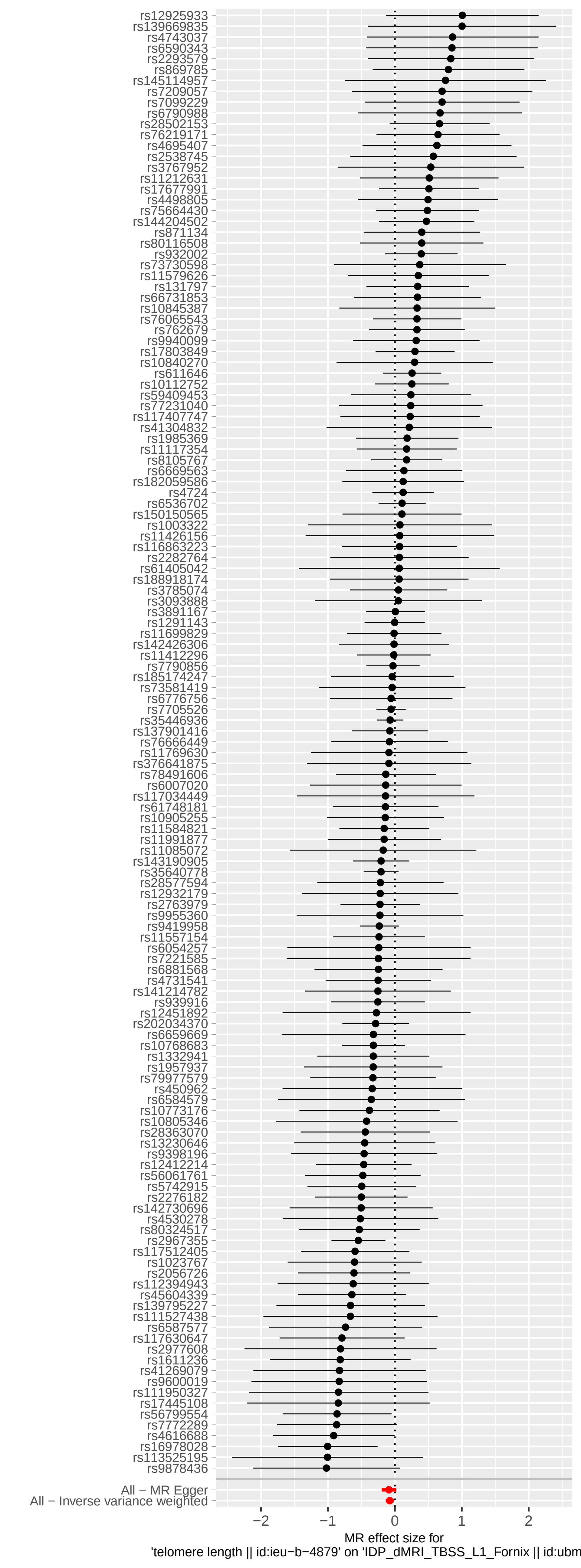
rs12925933
rs139669835
rs4743037
rs6590343
rs2293579
rs869785
rs145114957
rs7209057
rs7099229
rs6790988
rs28502153
rs76219171
rs4695407
rs2538745
rs3767952
rs11212631
rs17677991
rs4498805
rs75664430
rs144204502
rs871134
rs80116508
rs932002
rs73730598
rs11579626
rs131797
rs66731853
rs10845387
rs76065543
rs762679
rs9940099
rs17803849
rs10840270
rs611646
rs10112752
rs59409453
rs77231040
rs117407747
rs41304832
rs1985369
rs11117354
rs8105767
rs6669563
rs182059586
rs4724
rs6536702
rs150150565
rs1003322
rs11426156
rs116863223
rs2282764
rs61405042
rs188918174
rs3785074
rs3093888
rs3891167
rs1291143
rs11699829
rs142426306
rs11412296
rs7790856
rs185174247
rs73581419
rs6776756
rs7705526
rs35446936
rs137901416
rs76666449
rs11769630
rs376641875
rs78491606
rs6007020
rs117034449
rs61748181
rs10905255
rs11584821
rs11991877
rs11085072
rs143190905
rs35640778
rs28577594
rs12932179
rs2763979
rs9955360
rs9419958
rs11557154
rs6054257
rs7221585
rs6881568
rs4731541
rs141214782
rs939916
rs12451892
rs202034370
rs6659669
rs10768683
rs1332941
rs1957937
rs79977579
rs450962
rs6584579
rs10773176
rs10805346
rs28363070
rs13230646
rs9398196
rs12412214
rs56061761
rs5742915
rs2276182
rs142730696
rs4530278
rs80324517
rs2967355
rs117512405
rs1023767
rs2056726
rs112394943
rs45604339
rs139795227
rs111527438
rs6587577
rs117630647
rs2977608
rs1611236
rs41269079
rs9600019
rs111950327
rs17445108
rs56799554
rs7772289
rs4616688
rs16978028
rs113525195
rs9878436
All − MR Egger
All − Inverse variance weighted
−2
−1
0
1
2
MR effect size for
'telomere length || id:ieu−b−4879' on 'IDP_dMRI_TBSS_L1_Fornix || id:ubm
